# Exosomal microRNA *miR-1246* induces cell motility and invasion through the regulation of *DENND2D* in oral squamous cell carcinoma

**DOI:** 10.1038/srep38750

**Published:** 2016-12-08

**Authors:** Sujata Sakha, Tomoki Muramatsu, Koji Ueda, Johji Inazawa

**Affiliations:** 1 Department of Molecular Cytogenetics, Medical Research Institute, Tokyo Medical and Dental University, Tokyo, Japan; 2 Project for Personalized Cancer Medicine, Genome Center, Japanese Foundation for Cancer Research, Tokyo, Japan; 3 Bioresource Research Center, Tokyo Medical and Dental University, Tokyo, Japan

## Abstract

Metastasis is associated with poor prognosis in cancers. Exosomes, which are packed with RNA and proteins and are released in all biological fluids, are emerging as an important mediator of intercellular communication. However, the function of exosomes remains poorly understood in cancer metastasis. Here, we demonstrate that exosomes isolated by size-exclusion chromatography from a highly metastatic human oral cancer cell line, HOC313-LM, induced cell growth through the activation of ERK and AKT as well as promoted cell motility of the poorly metastatic cancer cell line HOC313-P. MicroRNA (miRNA) array analysis identified two oncogenic miRNAs, *miR-342–3p* and *miR-1246*, that were highly expressed in exosomes. These miRNAs were transferred to poorly metastatic cells by exosomes, which resulted in increased cell motility and invasive ability. Moreover, *miR-1246* increased cell motility by directly targeting DENN/MADD Domain Containing 2D (*DENND2D*). Taken together, our findings support the metastatic role of exosomes and exosomal miRNAs, which highlights their potential for applications in miRNA-based therapeutics.

Oral cancer is a subtype of head and neck cancer (HNC) that arises on the lip, tongue or floor of the mouth^1^. Oral cancer is the sixth most common cancer diagnosed worldwide and was the cause of more than 145,000 deaths in 2012, of which 77% occurred in less developed regions^2^. Ninety percent of oral cancers are squamous cell carcinomas, which display high rates of lymph node metastasis^3^. Tobacco and alcohol are major risk factors for oral cancer in developing countries, and recent studies have implicated Human Papilloma Virus (HPV) infection as another risk factor in developed countries^1^. Although early stage HNCs are associated with high cure rates through surgery and radiotherapy, up to 50% of oral cancer patients present with advanced disease, secondary tumors and metastasis^3^. Importantly, metastasis accounts for most deaths in oral cancer patients and poses a major challenge in the treatment of oral squamous cell carcinoma (OSCC)^3^. Activating invasion and metastasis, which is one of the six hallmarks of cancer, is a complex multistep process in human tumor pathogenesis^4^,^5^. The molecular pathogenesis behind the development of such metastatic advances is an area of intense research, and recent study on the role of extracellular vesicles called exosomes has shown promise in explaining metastasis^6^.

Exosomes are 30–100-nm-diameter vesicles of endosomal origin that are secreted in all biological fluids, including blood, urine, saliva, cerebrospinal fluid and *in vitro* cell culture medium^6^,^7^,^8^. These vesicles form part of an intercellular communication system, which makes them potentially useful for therapy as well as biomarkers of diseases, such as cancer^6^,^9^. Recent reports have suggested that the RNA signature of urinary exosomes can serve as a clinical diagnostic biomarker of prostate cancer risk in patients^10^. In another study, the authors found that exosomes from tumors drive the formation of the pre-metastatic niche and determine organotropic metastasis through the integrins of exosomes^11^. Exosomes play such biological and pathological roles in intercellular communication through their cargo molecules, which includes protein and genetic material, such as microRNA (miRNA)^12^,^13^. MicroRNAs are small non-coding RNAs that mediate destabilization and/or translational repression of target messenger RNA (mRNA) molecules and thus reduce the final protein output. An increasing amount of direct evidence has linked miRNAs to cancer development and progression^14^,^15^. MicroRNAs upregulated in some cancers that promote oncogenesis by targeting tumor suppressor genes are known as “oncogenic miRNAs (oncomiRs)”, whereas downregulated miRNAs are known as “tumor suppressor miRNAs (TS-miRNAs)”^16^. MicroRNAs can also be packaged into the multivesicular bodies and released as exosomes into the extracellular environment^17^. Despite many studies on exosomes function, the exact molecular basis behind the biological and pathological function of exosomes is poorly understood.

We previously established the highly metastatic oral cancer subline HOC313-LM from the HOC313 parent cell line (HOC313-P) and we used these cell lines to study the function of exosomes in cancer progression^18^. Our results revealed that exosomes containing miRNA cargo derived from the highly metastatic HOC313-LM cells are one of the factors that promote cell growth, migration and invasion of HOC313-P cells, which can increase the malignant potential of the parental cell line.

## Results

### LM-exosomes can be isolated by size-exclusion chromatography

**Figure 1:**
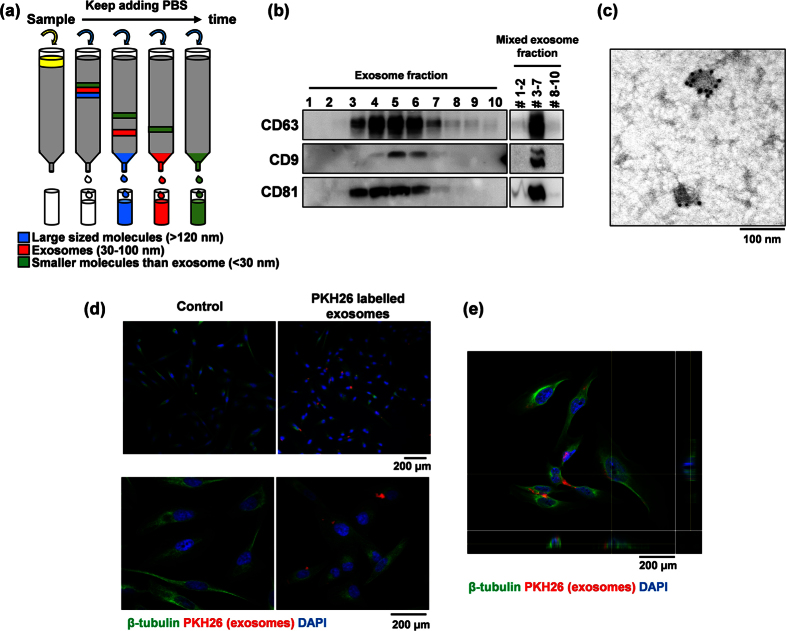
LM-exosomes are isolated by size-exclusion chromatography. (**a**) Schematic diagram of size-exclusion chromatography, where an aliquot of 400 μl of culture medium filtered by centrifugation was passed through a Sepharose column, and 10 consecutive 100-μl fractions were collected by PBS washes. Larger molecules were collected in the initial fractions, followed by smaller molecules. (**b**) The expression of exosomal biomarkers was analyzed by western blotting all 10 fractions (left) as well as by western blotting pooled fractions (right). (**c**) Characterization of LM-exosomes by immunogold-TEM. Vesicles isolated from the culture medium of HOC313-LM cells were positive for the exosomal marker CD63. (**d**) Fluorescence microscopy analysis of PKH26-labeled LM-exosomes (red) taken up by HOC313-P cells after 14 hours of incubation with the exosomes. Bar, 200 μm. (**e**) 3D confocal microscopy analysis confirms the incorporation of exosomes within the cellular compartment. (Red: exosomes, Green: β-tubulin, Blue: DAPI) Bar, 200 μm.

We previously established a highly metastatic human OSCC subline (HOC313-LM) from HOC313 parental cells (HOC313-P)^18^. To investigate the significance of exosome in the metastatic capacity of HOC313-LM cells, we isolated and characterized exosomes from the culture media of HOC313-LM cells using size-exclusion chromatography and western blotting analysis. Size-exclusion chromatography can be used for exosomes isolation to acquire exosomes devoid of small plasma protein contaminants (Fig. 1a)^19^. To evaluate the efficiency of exosomes purification using this method, we characterized the exosomes by western blotting and transmission electron microscope (TEM) analysis. The most widely accepted tetraspanin markers of exosomes, CD9, CD63 and CD81, could be detected in consecutive fractions three through seven (Fig. 1b). We combined the isolated fractions into three groups containing fractions 1–2, fractions 3–7 and fractions 8–10, and we found that fractions 3–7 showed the strongest expression of exosome markers, which suggests exosomes enrichment in fractions 3–7. TEM analysis also demonstrated the presence of exosomes in fractions 3–7 (Fig. 1c). Therefore, we defined fractions 3–7 as HOC313-LM-exosomes (LM-exosomes).

To visualize the uptake of LM-exosomes by HOC313-P cells, we labeled LM-exosomes with PKH26, a red fluorescent dye, and added the LM-exosomes to HOC313-P cells in culture. PKH26 dye contains long aliphatic tails that are incorporated into the lipid membrane of exosomes^20^. After 14 hours of treatment with labeled LM-exosomes, we found that HOC313-P cells acquired positive PKH26 signal compared with control cells (Fig. 1d, e). These observations suggest that LM-exosomes isolated by size-exclusion chromatography could be effectively taken up by HOC313-P cells.

### LM-exosomes induce cell growth and increase the migration and invasion ability of HOC313-P cells

**Figure 2:**
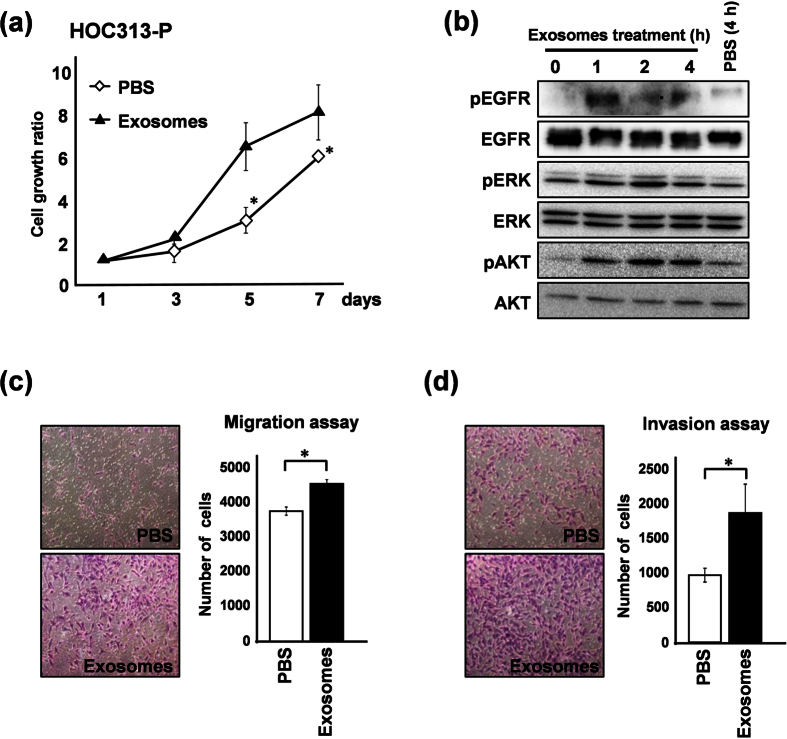
LM-exosomes increase cell viability and cell motility in HOC313-P cells. (**a**) HOC313-P cells were treated with PBS or LM-exosomes, and the effect of exosomes on cell growth was determined using an *in vitro* WST-8 assay at the indicated times. (**b**) Western blot analysis of EGFR, ERK and AKT and their phosphorylation status is shown in HOC313-P cells treated with LM-exosomes for the indicated amounts of time. (**c** and **d**) Cell migration was assessed by Transwell migration assay (**c**) and cell invasion was assessed by Transwell invasion assay (**d**) in HOC313-P cells treated with either PBS or LM-exosomes. Experiments were performed in triplicate. (Bars, SD). Student’s *t*-test was used for statistical analysis; asterisks represent *P* < 0.05 versus each control transfectant.

To determine the biological significance of secreted LM-exosomes, we performed a functional assay of LM-exosomes in HOC313-P cells. *In vitro* cell growth was assessed in HOC313-P cells treated with LM-exosomes for seven consecutive days. A cell growth assay showed that HOC313-P cells treated with LM-exosomes proliferated rapidly compared to the control group (Fig. 2a). In contrast, the cell growth rate of HOC313-LM cells did not change upon treatment with LM-exosomes (Supplementary Fig. S2). To define the mechanism by which LM-exosomes promote cell growth in HOC313-P cells, we examined the phosphorylation status of cell survival signaling proteins EGFR, ERK and AKT. Notably, the phosphorylation status of these proteins was elevated in HOC313-P cells upon treatment with LM-exosomes (Fig. 2b). In addition, we observed that the phosphorylation levels of ERK and AKT in HOC313-LM cells were higher than those of HOC313-P cells (Supplementary Fig. S2).

We next assessed the migration and invasion ability of HOC313-P cells in the presence of LM-exosomes. The number of cells that migrated and invaded increased upon LM-exosome treatment compared with the control group (Fig. 2c,d). Moreover, we found that the migration and invasion rates of HOC313-LM cells were not changed upon treatment with LM-exosomes, which is consistent with the increased cell growth rate of these cells (Supplementary Fig. S2). Collectively, these data suggest that LM-exosomes can promote the cell growth, migration and invasion ability of HOC313-P cells.

### LM-exosomes contain oncomiRs *miR-342–3p* and *miR-1246* that are transferred to HOC313-P cells for intercellular communication

**Figure 3:**
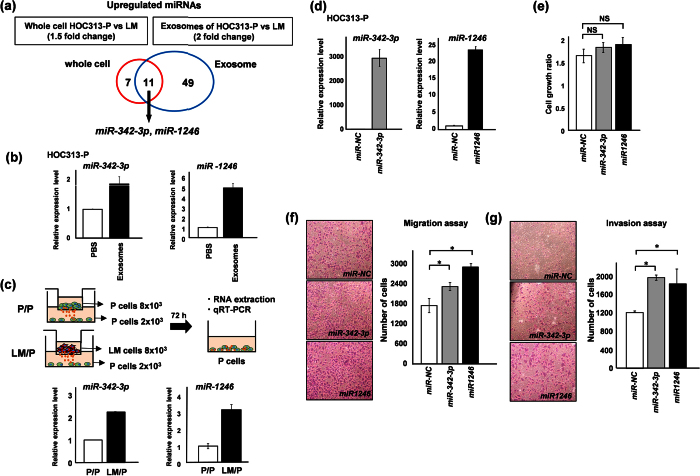
LM-exosomes contain oncogenic miRNAs and mediate their transfer to promote intercellular communication. (**a**) The Venn diagram illustrates the overlapping result of differentially expressed miRNAs in whole cells and exosomes, including HOC313-P and HOC313-LM cell comparisons. Two miRNAs, *miR-342-3p* and *miR-1246*, were upregulated in both whole cells and exosomes. (b and c) *MiR-342-3p* and *miR-1246* were transferred to HOC313-P cells treated with LM-exosomes (**b**) or across a Transwell membrane through exosomes (**c**). (**d**) Validation of *miR-342-3p* and *miR-1246* expression after each miRNA transfection in HOC313-P cells was measured by qRT-PCR. MicroRNA expression levels were normalized to *RNU6B* expression. MicroRNA expression levels of *miR-342-3p* (left) and *miR-1246* (right) relative to the negative controls are shown. (**e**) Cell growth after 72 hours of transfection with *miR-342-3p, miR-1246* and *miR-NC* in HOC313-P cells was evaluated by WST-8 assay. (**f** and **g**) Cell motility was assessed by Transwell migration assay (**f**) and cell invasion was assessed by Transwell invasion assay (**g**) in HOC313-P cells transfected with *miR-342-3p* or *miR-1246*. Experiments were performed in triplicate. (Bars, SD). Student’s *t*-test was used for statistical analysis; asterisks represent *P* < 0.05 versus each control transfectant.

RNA and proteins contained in exosomes contribute to exosome function^21^. To investigate the oncogenic functions of LM-exosomes through their miRNA content, we first extracted RNA from HOC313-P and -LM cells and their respective exosomes. Bioanalyzer results showed that exosomes were highly enriched in small RNA species compared with cellular RNA (Supplementary Fig. S1). We then performed miRNA array analysis using whole RNA from HOC313-P and -LM cells and their respective exosomal RNA (Fig. 3a). When we analyzed the miRNA array data for miRNAs that are upregulated in HOC313-LM cells, we found that 18 miRNAs were highly expressed in HOC313-LM cells compared with HOC313-P cells. Interestingly, more miRNAs (60) were expressed at higher levels in LM-exosomes when compared with HOC313-P-derived exosomes. The most salient finding was a set of 11 miRNAs that are commonly upregulated in HOC313-LM cells at the cellular and exosomal level (Table 1). Based on these results, we focused on seven miRNAs, *miR-17, miR-30a-3p, miR-30a-5p, miR-92a, miR-181a, miR-342–3p* and *miR-1246*, all of which have been reported as oncogenic miRNAs (oncomiRs)^22^,^23^,^24^,^25^,^26^,^27^,^28^. Using quantitative RT-PCR (qRT-PCR), we could validate the differential expression of six of these miRNAs (all except *miR-92a*) in cells and exosomes (Supplementary Fig. S3). To evaluate whether these six miRNAs could affect cell growth, we performed a cell growth assay and found that *miR-342–3p* and *miR-1246* did not inhibit cell growth (Fig. 3e, Supplementary Fig. S3). Therefore, we focused on the functions of *miR-342–3p* and *miR-1246*. These data suggest that a specific miRNA population could be selectively sorted into the exosomes, of which *miR-342–3p* and *miR-1246* could act as oncomiRs in oral squamous cell carcinoma.

### Exosomes mediate transfer of miRNAs to adjacent or distant cells for intercellular communication

To determine whether exosomes mediate intercellular transfer of miRNAs, we incubated HOC313-P cells with LM-exosomes. After 72 hours of treatment with LM-exosomes, qRT-PCR analysis revealed that the levels of *miR-342–3p* and *miR-1246* in HOC313-P cells were elevated compared with control cells (Fig. 3b). In addition, to examine whether *miR-342–3p* and *miR-1246* transfer requires direct cell contact, we set up a Transwell assay in which HOC313-P cells or HOC313-LM cells were separated from HOC313-P cells by a porous 1-μm upper membrane in a ratio of 4:1. Under this condition, only acellular material, such as exosomes or other soluble factors, can migrate across these membranes^13^. Seventy-two hours later, the HOC313-P cells in the bottom chamber were analyzed. Consistent with the miRNA transfer observed in LM-exosome-treated cells, the expression levels of *miR-342* and *miR*-*1246* increased when HOC313-LM cells were in the upper chamber relative to when HOC313-P cells were in the upper chamber (Fig. 3c). Therefore, LM-exosomes may selectively mediate transfer of their contents into the recipient cells at adjacent or distant sites and could induce an oncogenic outcome in the recipient cells.

### Exosomal miRNAs *miR-342-3p* and *miR-1246* increase cell motility but not cell growth in HOC313-P, TSU and HeLa cells

Following the observation that LM-exosomes function in an oncogenic manner in HOC313-P cells, we hypothesized that the miRNA cargo of LM-exosomes was responsible for such biological functions. To test the functions of *miR-342-3p* and *miR-1246*, we transiently transfected *miR-342–3p* and/or *miR-1246* into HOC313-P, TSU and HeLa cells. We confirmed the level of miRNA expression by qRT-PCR analysis and found that these miRNAs were overexpressed compared with control-transfected HOC313-P, TSU and HeLa cells (Fig. 3d, Supplementary Fig. S4, S5). Cell growth was not significantly affected by transient transfection with either miRNA in HOC313-P, TSU and HeLa cells (Fig. 3e, Supplementary Fig. S4, S5). These results suggest that the growth-promoting ability of LM-exosomes might not be dependent on these miRNAs but rather on other cargo molecules of LM-exosomes. We also examined the migration and invasion ability of HOC313-P, TSU and HeLa cells by overexpressing *miR-342–3p* and/or *miR-1246*. Overexpression of *miR-342–3p* and *miR-1246* enhanced the migration and invasion ability in HOC313-P, TSU and HeLa cells (Fig. 3f, g, Supplementary Fig. S4, S5). Taken together, these data suggest that *miR-342–3p* and *miR-1246* delivered via LM-exosomes act as oncomiRs by affecting the cell motility of the recipient cells.

### Exosomal *miR-1246* directly and functionally targets the *DENND2D* gene

**Figure 4:**
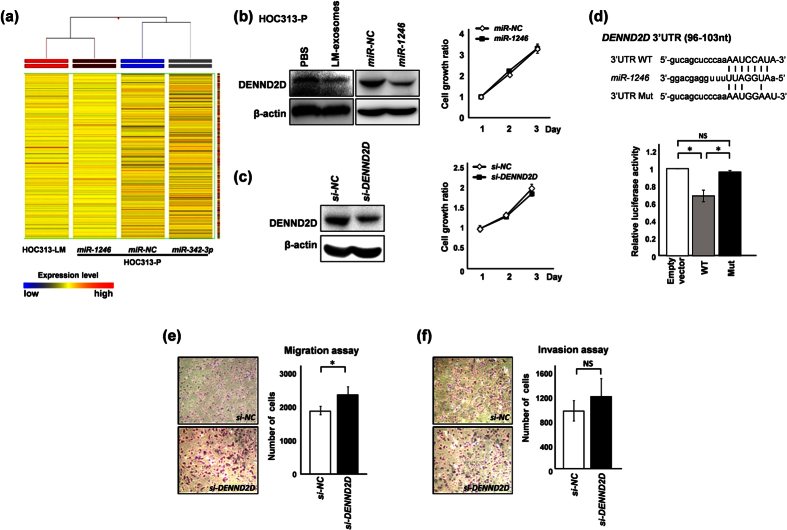
Overexpression of *miR-1246* induces a highly metastatic phenotype based on gene expression pattern and regulates cell motility by directly targeting *DENND2D*. (**a**) Unsupervised hierarchal clustering analysis in HOC313-LM, *miR-342-3p-, miR-1246-* and *miR*-*NC*-transfected HOC313-P cells using gene expression microarray data was performed. Each horizontal colored bar represents one probe set, and the color of the bar indicates the degree of expression (Red: high expression, Blue: low expression). (**b** and **c**) Downregulation of DENND2D at the protein level was confirmed by western blot analysis after treatment with LM-exosomes and transfection of *miR-1246* (**b**) or *DENND2D*-specific siRNA (**C**). Cell growth 24–72 hours after transfection of *miR-1246* (**b**) or *DENND2D*-specific siRNA (**c**) was assessed by WST-8 assay in HOC313-P cells. (**d**) Luciferase reporter assay of HOC313-P cells co-transfected with a luciferase reporter plasmid containing a wild-type (WT) or mutant (Mut) *DENND2D* 3′UTR and either *miR-NC* or *miR-1246*. Seed sequences of *miR-1246* within the *DENND2D* 3′UTR and mutant sequences are indicated (left). (e and f) Cell motility was assessed by Transwell migration assay (**e**) and cell invasion was assessed by Transwell invasion assay (**f**) in HOC313-P cells subjected to *DENND2D* knockdown or *siControl* transfection. Each data point represents the mean of three experiments (bars, SD). Asterisks represent *P* < 0.05 versus a control transfectant. NS: no significance.

To gain insight into the mechanism through which exosomal miRNAs promote the migration and invasion of HOC313-P cells, we analyzed the genes that are downregulated by these miRNAs. To this end, we performed gene expression array analysis on HOC313-LM cells and on *miR-NC-, miR-342–3p-* or *miR-1246*-transfected HOC313-P cells. The array data showed that the gene expression pattern upon *miR-1246* transfection was similar to the HOC313-LM cell gene expression pattern (Fig. 4a). To identify target genes of *miR-1246*, we focused on 13 genes that were downregulated upon *miR-1246* transfection according to the array data (Table 2). We selected three candidate genes based on previously described roles as tumor suppressors and their identification through the TargetScan program (www.targetscan.org) as predicted target genes of *miR-1246*^29^,^30^,^31^. Among the candidate genes, we found that *DENN/MADD Domain Containing 2D (DENND2D*) was downregulated 6.2-fold in *miR-1246*-transfected cells compared with control-transfected cells. Protein expression of DENND2D was reduced upon treatment with LM-exosomes and/or transfection of *miR-1246* in HOC313-P, TSU and HeLa cells (Fig. 4b, Supplementary Fig. S4, S5). In addition, we performed a cell growth assay in *miR-1246*-overexpressing cells and observed that cell growth was not affected by *miR-1246* expression (Fig. 3e, 4b, Supplementary Fig. S4, S5). To evaluate the functional consequences of *DENND2D*, we suppressed *DENND2D* expression in HOC313-P, TSU and HeLa cells using *DENND2D*-specific siRNA and assessed the cell growth of these cells (Fig. 4c, Supplementary Fig. S4, S5). Cell growth was not affected by the suppression of *DENND2D*, which is consistent with our results analyzing cell growth upon *miR-1246* or *DENND2D*-specific siRNA transfection (Fig. 4b,4c).

To determine whether *DENND2D* is a direct target of *miR-1246* binding to the *miR-1246* seed sequence in the 3′UTR, we performed a luciferase assay in HOC313-P cells using a reporter plasmid vector containing either a wild-type (WT) or mutant (Mut) seed sequence within the 3′UTR (Fig. 4d). Our results showed a 35% reduction in luciferase activity for the WT vector compared with empty vector, whereas the luciferase activity was completely restored upon expression of the mutant vector, which contains an insertion mutation within the seed sequence (Fig. 4d). These results suggest that *miR-1246* can downregulate the expression of *DENND2D* by direct binding to the 3′UTR.

Furthermore, knockdown of *DENND2D* resulted in an increase in HOC313-P, TSU and HeLa cells motility (Fig. 4e, Supplementary Fig. S4, S5). In addition, when *DENND2D* was knocked down, cells showed increased invasion ability compared with the control group (Fig. 4f, Supplementary Fig. S4, S5). Collectively, these results suggest that *DENND2D* is regulated by *miR-1246*, and upon suppression, *DENND2D* promotes the migration and invasion of HOC313-P, TSU as well as HeLa cells.

## Discussion

Since the initial studies showing that exosomes can mediate the intercellular transfer of RNA and proteins, many studies have focused on the contents of exosomes involved in intercellular communication^17^. In the present study, we identified functional oncogenic miRNAs that can be delivered from the highly metastatic oral cancer cell line HOC313-LM to the poorly metastatic cell line HOC313-P through exosomes. Notably, exosomal *miR-342–3p* and *miR-1246* induced a pro-metastatic phenotype, including increased cell motility and invasion, and *miR-1246* directly targets *DENND2D* expression by binding to its 3′UTR. The suppression of *DENND2D* promoted the migration and invasion ability of HOC313-P, TSU and HeLa cells, which is consistent with the role of LM-exosomes in promoting HOC313-P cell migration and invasion. However, an intriguing remaining question is which of the exosomal constituents is/are responsible for the increased cell growth induced by LM-exosomes.

Despite progress in exosome research, no standard methods exist for providing exact quantitative and qualitative analysis of exosomes^6^,^32^. Some methods have been developed for the isolation of exosomes, including ultracentrifugation, commercial-based kits and size-exclusion chromatography. In the present study, we chose size-exclusion chromatography to isolate exosomes^19^ because we could purify the most RNA from exosomes isolated using this method compared with other methods (Supplementary Fig. S6). In addition, comparative studies showed that isolation of exosomes by size-exclusion chromatography retains the biophysical properties of exosomes, resulting in higher yield^33^.

We found that exosomes isolated from HOC313-LM cells could induce malignant features in HOC313-P cells by increasing their cell growth and cell motility. Cell migration and invasion are key features of metastasis^4^,^5^. Many studies have assessed the importance of exosomes in tumor metastasis; for example, one study showed that exosomes isolated from highly metastatic melanomas increased the metastatic potential of primary tumors by inducing bone marrow progenitor cells to acquire a pro-metastatic phenotype^34^. A more recent study reported that exosomal *miR-181c* promoted the destruction of the blood-brain barrier by altering actin dynamics through the downregulation of 3-phosphoinositide-dependent protein kinase-1 (*PDPK1*) and induced brain metastases in cancer patients^35^. Thus, the exosomes play a role in the establishment of the metastatic niche by communication between cancer cells and normal cells^34^. Moreover, our data indicate that exosomes secreted by highly metastatic cells contribute to the cancer progression of poorly metastatic cells, which is consistent with the idea that cancer cells interact with each other through exosomes.

The recent discovery of extracellular miRNAs has made apparent their role in intercellular communication and metastatic potential of cancer cells^36^. In the present study, the miRNA microarray analysis of exosomal and cellular RNA showed that the secretion of miRNAs in exosomes is selective and does not necessarily correlate with their abundance in the cell of origin. Thus, these results suggest the existence of a selective mechanism to enrich exosomes with miRNAs that are not abundant in their cell of origin. Moreover, the oncomiRs *miR-342–3p* and *miR-1246* were significantly enriched in exosomes compared with other intracellular compartments and were responsible for the promotion of the metastatic potential of HOC313-P cells. MicroRNAs taken up by HOC313-P cells showed almost no difference when comparing direct treatment of LM-exosomes and indirect Transwell co-culture (Fig. 3b and c). This observation underscores the potency of the exosomal delivery mechanism and suggests that uptake occurs at both local sites and distant sites to increase metastatic potential. Although migration and invasion abilities of HOC313-P, TSU and HeLa cells were increased upon overexpression of *miR-342–3p* and/or *miR-1246*, we did not observe any significant effect on cell growth rates. Thus, we propose that the cell proliferation-inducing activity of exosomes is likely governed by mechanisms other than miRNAs, such as cytokines or other exosomal components, that have yet to be discovered^37^.

*MicroRNA 342–3p* plays dual roles in tumor suppression and tumor promotion^25^,^38^. A heatmap of the gene expression profiles of *miR-342–3p-, miR-1246-*, and *miR-NC-*transfected HOC313-P cells and the highly metastatic HOC313-LM cell line revealed that transfection with *miR-342–3p* is insufficient to induce a HOC313-LM-like gene expression profile, in contrast to that of *miR-1246* (Fig. 4a). Therefore, we excluded *miR-342–3p* from further investigation and continued exploring the functional mechanism of *miR-1246*. Recently, extracellular *miR-1246* has been reported to promote lung cancer proliferation and enhance radioresistance^27^,^28^. Importantly, in our study, we found that *DENND2D* is a direct target of *miR-1246* and demonstrated a gene expression pattern that resembled that of HOC313-LM cells (Fig. 4a). *DENND2D* is a tumor suppressor gene but is only known to be downregulated by promoter hypermethylation in gastric and hepatic cancers^31^,^39^. Our results show for the first time that *DENND2D* is also regulated by miRNAs and can be directly targeted and downregulated by *miR-1246*. We found that inhibition of *DENND2D* could induce cell migration and invasion in HOC313-P, TSU and HeLa cells but not cell growth. These findings are consistent with a recent report in which *miR-522* expression induce cell motility by targeting *DENND2D* in lung cancer^40^. Hence, downregulation of *DENND2D* by *miR-1246* results in the acquisition of migratory and invasive abilities of poorly metastatic cancer cells in various types of cancer.

Overall, our study demonstrates an important role for cancer cell-derived exosomes and exosomal miRNAs in cancer progression. Interestingly, we show evidence that the abundance of oncogenic *miR-342–3p* and *miR-1246* in cancer exosomes is significantly associated with malignancy. One of the molecular mechanisms resulting in cancer progression involves the direct targeting of *DENND2D* by *miR-1246*. In addition to important cellular functions, secreted exosomal miRNAs could serve as therapeutic targets for novel cancer treatments as well as diagnostic biomarkers.

## Materials and Methods

### Cell culture

The human oral cancer cell line HOC313 was generously donated by Dr. T. Amagasa (Tokyo Medical and Dental University, Japan). The highly metastatic HOC313-LM cell line was previously derived from HOC313 cells by our laboratory^18^. These cells, TSU cells, an oral cancer cell line and HeLa cells, a cervical cancer cell line, were maintained in Dulbecco’s Modified Eagle Medium (DMEM) containing 10% fetal bovine serum (FBS). Cells used for exosome isolation were cultured in DMEM supplemented with 10% exosome-depleted FBS. Exosome-depleted FBS was prepared by ultracentrifugation. First, FBS was centrifuged at 2,000 × *g* for 15 minutes at 4 °C followed by a second centrifugation step of the supernatant at 12,000 × *g* for 35 minutes at 4 °C. Then, the supernatant was subjected to ultracentrifugation at 110,000 × *g* for 16 hours at 4 °C (70, 70 Ti, 38,600 rpm Beckman rotor model). Leaving the exosome pellet undisturbed, the clear supernatant was carefully collected and filtered through a syringe filter (0.22-μm pore size, Merck Millipore, Damstadt, Germany).

### Isolation of exosomes

For size-exclusion chromatography, Sepharose beads (Sephacryl S-400 High Resolution, 17–0609–10, GE Healthcare, Buckinghamshire, UK) equilibrated with PBS (80% slurry) were packed into a 5-ml column (GE Healthcare) to a final volume of 3.6 ml. Cells were cultured for 72 hours in DMEM supplemented with 10% exosome-depleted FBS, and the supernatants were collected in 15-ml Amnicon Centrifugal filter units (UFC 901024) (Merck Millipore) after passing through a 0.45-μm syringe filter (Merck Millipore). The sample was then centrifuged at 5,000 × *g* for 70 minutes at 4 °C to remove cellular debris and unwanted proteins. A volume of 400 μl of the centrifuged sample was loaded onto the packed column and was washed with 800 μl of PBS. Exosomes were then eluted in 10 consecutive 100-μl fractions of PBS by isocratic gravity-driven flow. The ultracentrifugation method of exosome isolation was conducted as described elsewhere^11^. The Total Exosome Isolation Reagent (from cell culture media) kit #44578259 (Thermo Fisher Scientific, CA, USA) was used for the kit method of exosome isolation according to the protocol recommended by the manufacturer.

### Negative staining immunogold TEM of LM-exosomes

Exosomes isolated from cell culture medium were placed on nickel grids for 30 min at room temperature (RT) and were fixed in buffer (4% paraformaldehyde in 0.1 M Tris-HCl buffer [pH 7.4]) for 20 min at RT after removing the solution on the nickel grids. The nickel grids were then washed six times with 0.1 M Tris-HCl buffer (pH 7.4) and were blocked with 0.1 M glycine-Tris (pH 7.4) for 10 min at RT. After washing with 12 mM Na-P buffer, the nickel grids were blocked with 3% goat serum/0.1 M Tris-HCl buffer (pH 7.4) for 20 min at RT. The nickel grids were incubated overnight at 4 °C with anti-CD63 antibody (#556019, BD Biosciences, Franklin Lakes, NJ) diluted with buffer A (0.1 M Tris-HCl buffer [pH 7.4], 0.1 M PB) 1:20, followed by incubation with mouse anti-mouse IgG (10 nm gold colloidal particles, British Bio Cell International, Golden Gate, UK) diluted with 0.1 M Tris-HCl buffer (pH 7.4) 1:25 for 1 hour at RT. After washing with 0.1 M Tris-HCl buffer (pH 7.4) containing 0.1 M PB, the nickel grids were fixed with 2.5% GA in 0.1 M PB for 10 min at RT and were further washed with 0.1 M PB in Distilled Water. Finally, the grids were embedded in 1.5% uranyl acetate for 2 min at RT.

### PKH26 labeling of exosomes

To track exosome internalization, exosomes were fluorescently labeled using PKH26 (MINI26–1KT), a red membrane dye (Sigma-Aldrich, St. Louis, MO, USA), according to the protocol recommended by the manufacturer, with minor modifications. Briefly, 300 μl of exosomes was suspended into 100 μl of Diluent C. Separately, Diluent C was mixed with 1.4 μl of PKH26. Immediately the exosome solution was mixed with stain solution and was incubated for 4 minutes. The labeling reaction was stopped by adding an equal volume of 1% BSA (700 μl). The labeled exosomes were added to the HOC313-P cells and were incubated at 37 °C for 14 hours, after which the cells were fixed with 2% formaldehyde and were visualized by immunofluorescence microscopy.

### Cell growth, migration and invasion assay

The number of viable cells at various time points after exosome treatment and miRNA transfection were assessed using a colorimetric water-soluble tetrazolium salt (WST-8) assay, as described elsewhere^41^. Transwell migration and invasion assays were conducted in 24-well modified chambers pre-coated without (migration) or with (invasion) Matrigel (BD BioCoat, BD Biosciences), as described elsewhere^41^.

Exosomes from HOC313-LM cells (5 μg) were added to the upper chamber and were incubated for 24 hours to allow cell migration through the membrane. Migratory and invasive cells were fixed with a Diff-Quik kit (Sysmex, Hyogo, Japan) according to the protocol recommended by the manufacturer. Five fields were counted per well, and the average number of migrated and invaded cells per field was calculated.

### Transwell co-culture assay

HOC313-LM cells were plated in 1-μm porous Transwell inserts (Corning, Corning, NY, USA) with the upper chamber hanging over the lower chamber, which contained HOC313-P cells. The ratio of cells in the upper chamber to cells in the lower chamber was 4:1 (HOC313-LM: HOC313-P). The cells were co-cultured for three days. In control wells, both chambers were plated with HOC313-P cells at the same ratio of 4:1.

### Western blotting

Cells were harvested, and proteins were isolated from cells, as described elsewhere^20^. Exosomes were directly used for protein analysis. The protein concentration of cells and exosomes was determined using a protein assay kit (Bio-Rad, Hercules, CA, USA), and samples were separated on SDS polyacrylamide gels for western blotting analysis. An anti-CD9 antibody (EPR2949) was purchased from Abcam (Cambridge, UK), anti-CD63 antibody #556019 was purchased from BD Biosciences (San Joes, CA USA), anti-CD81 antibody (CBL579) was purchased from EMD-Millipore, anti-EGFR antibody (#sc-03) was purchased from Santa Cruz Biotechnology (Santa Cruz, CA, USA), anti-phospho-EGFR (#2234), anti-AKT (#9272 S), anti-phospho-AKT (#9271 S), anti ERK (#9102 S) and anti-phospho-ERK (#9106 S) antibodies were purchased from Cell Signaling Technologies (Danvers, MA, USA) and anti-β-actin antibody was purchased from Sigma-Aldrich.

### RNA isolation and quantitative RT-PCR (qRT-PCR)

Total RNA from cells was extracted using the acid guanidinium thiocyanate-phenol-chloroform extraction method, and total RNA from exosomes was extracted using a Total Exosome RNA and Protein Isolation kit (Thermo Fisher Scientific) according to the instructions recommended by the manufacturer. Isolated RNA was screened for purity and concentration using a Nanodrop-1000 Spectrophotometer (Thermo Fisher Scientific). An Agilent 2100 Bioanalyzer (Agilent Technologies, Santa Clara, CA, USA) for total RNA (RNA pico chips, Agilent Technologies) was used to assess the large and small RNA profiles isolated from cells and exosomes. Quantitative RT-PCR (qRT-PCR) of miRNA was performed using an ABI7500 instrument (Applied Biosystems; Foster City, CA, USA) using primers purchased from Applied Biosystems. Briefly, 10 ng of total RNA was reverse-transcribed using a Taqman Reverse Transcription kit followed by qRT-PCR performed using a custom Taqman miRNA Assay kit (Applied Biosystems). The miRNA concentration was normalized to the endogenous control RNU6B. The following primers were used for the Taqman assay: human *miR-17* (002308), *miR-30a-3p* (000416), *miR-30a-5p* (000417), *miR-92a* (002137), *miR-181a* (000480), *miR-342–3p* (002260), *miR-1246* (custom order) and *RNU6B* (001093).

### MicroRNA microarray analysis

Cellular and exosomal RNA was profiled for miRNA expression using a human miRNA V3 microarray, 8 × 15 K miRNA microarray system (Agilent Technologies) according to the instructions recommended by the manufacturer. The raw data were analyzed using GeneSpring GX10 software (Agilent Technologies).

### Gene expression array analysis

Analysis of gene expression profiles was performed using SurePrint G3 Gene Expression Microarrays (Agilent Technologies) according to the instructions recommended by the manufacturer. GeneSpring GX10 software (Agilent Technologies) was used to analyze the raw data.

### Luciferase reporter assay

Luciferase reporter plasmids were constructed by inserting the 3′UTR of *DENND2D* downstream of the luciferase gene within the pmirGLO Dual-Luciferase miRNA Target expression vector (Promega, Madison, WI). A site-specific mutation was created using the GeneTailor site-directed mutagenesis system (Thermo Fisher Scientific).

Forward primer (WT): TTCTCGAGGAATGACTAGAGCTACACACCA,

Reverse primer (WT): TTGTCGACTGAGCCCAGTTCTGTCAGCAT,

Forward primer (Mut): TGGAATTCCTCACTCTGAGTC,

Reverse primer (Mut): TTTTGGGAGCTGACAGTTTTGC.

Luciferase reporter plasmids were transfected into HOC313-P cells, and *miR-1246* or *miR-NC* was transfected five hours later. After 48 hours, firefly and renilla luciferase activities were measured using the Dual-Luciferase Reporter Assay system (Promega), and relative luciferase activity was calculated by normalizing the firefly luciferase reading with its corresponding internal renilla luciferase control, as described elsewhere^42^.

### Loss-of-function by small interfering RNA (siRNA) and gain-of-function by miRNA mimic

Loss-of-function was simulated by using siRNAs (*DENND2D* [L-016580-01-0005], *negative control* [D-001810-10-05]) purchased from GE Healthcare targeting the *DENND2D* gene transcript and a non-targeting construct used as a control. Gain-of-function was simulated by using miRNA mimics (*miR-342*-*3p* [MC12328], *miR-1246* [MC13182] and *negative control* [4464058]) purchased from Thermo Fisher Scientific. Each siRNA (20 nM) and each miRNA (20 nM) was transfected into HOC313-P or HOC313-LM cells using Lipofectamine RNAiMAX (Thermo Fisher Scientific) according to the instructions recommended by the manufacturer.

### Statistical analysis

Differences between subgroups were analyzed using Student’s *t*-test and were considered significant at a threshold of *P* < 0.05.

### Data deposition

The microarray data produced in our study have been submitted to the GEO database (http://www.ncbi.nlm.nih.gov/geo/) and have been assigned the identifier “GSE83981”.

## Additional Information

**How to cite this article**: Sakha, S. *et al*. Exosomal microRNA *miR-1246* induces cell motility and invasion through the regulation of *DENND2D* in oral squamous cell carcinoma. *Sci. Rep.* **6**, 38750; doi: 10.1038/srep38750 (2016).

**Publisher's note:** Springer Nature remains neutral with regard to jurisdictional claims in published maps and institutional affiliations.

## Supplementary Material

Supplementary Information

## Figures and Tables

**Table 1:** MicroRNA expression in whole cells and exosomes by miRNA microarray.

miRNA	Genomic Locus	Fold-change (whole cells)HOC313-LM vs. -P (1.5>)	Fold-change (exosomes) HOC313-LM vs.-P (2>
*hsa-let-7c*	21q21.1	2.705	3.138
*hsa-let-7d*	9q22.32	1.878	2.054
*hsa-miR-17*	13q31.3	1.971	2.877
*hsa-miR-30a*	6q13	2.667	6.611
hsa-miR-30a*	6q13	127.659	451.249
*hsa-miR-92a*	13q31.3	2.156	3.280
*hsa-miR-98*	Xp11.22	1.530	2.332
*hsa-miR-99a*	21q21.1	12.947	11.329
*hsa-miR-181a*	1q32.2	2.351	3.234
*hsa-miR-342-3p*	14q32.2	2.006	2.514
*hsa-miR-1246*	2q31.1	2.939	4.340

**Table 2:** Genes downregulated by *miR-1246* transfection in HOC313-P cells.

Gene		Genomic Locus	Fold-change *miR-1246* vs. NC (−4>)
*CCL5*	Chemokine (C-C Motif) Ligand	17q12	−9.373
*GUCY1A3*	Guanylate Cyclase 1, Soluble, Alpha 3	4q32.1	−7.383
*DENND2D*	DENN/MADD Domain Containing 2D	1p13.3–p13.2	−6.295
*SAA1*	Serum Amyloid A1	11p15.1	−6.072
*CAMK1G*	Calcium/Calmodulin-Dependent Protein Kinase IG	1q32.2	−5.133
*CXXC4*	CXXC Finger Protein 4	4q24	−4.825
*SAA2*	Serum Amyloid A2	11p15.1	−4.734
*COL6A4P2*	Collagen, Type VI, Alpha 4 Pseudogene 2	3q22.1	−4.561
*FLJ22447*	Uncharacterized LOC400221	14q23.1–q23.2	−4.437
*KCNQ1*	Potassium Channel, Voltage Gated KQT-Like Subfamily Q, Member 1	11p15.5	−4.371
*DTX4*	Deltex 4, E3 Ubiquitin Ligase	11q12.1	−4.238
*ELF3*	E74-Like Factor 3 (Ets Domain Transcription Factor, Epithelial-Specific)	1q32.1	−4.089
*MREG*	Melanoregulin	2q35	−4.067

## References

[b1] Kang H., Kiess A. & Chung C. H. Emerging biomarkers in head and neck cancer in the era of genomics. Nat Rev Clin Oncol 12, 11–26, doi: 10.1038/nrclinonc.2014.192 (2015). 25403939

[b2] Ferlay J. . Cancer incidence and mortality worldwide: sources, methods and major patterns in GLOBOCAN 2012. Int J Cancer 136, E359–386, doi: 10.1002/ijc.29210 (2015). 25220842

[b3] Rivera C. Essentials of oral cancer. Int J Clin Exp Pathol 8, 11884–11894 (2015). 26617944 PMC4637760

[b4] Hanahan D. & Weinberg R. A. Hallmarks of cancer: the next generation. Cell 144, 646–674, doi: 10.1016/j.cell.2011.02.013 (2011). 21376230

[b5] Giancotti F. G. Mechanisms governing metastatic dormancy and reactivation. Cell 155, 750–764, doi: 10.1016/j.cell.2013.10.029 (2013). 24209616 PMC4354734

[b6] Andaloussi E. L., Mäger S.I., Breakefield X. O. & Wood M. J. Extracellular vesicles: biology and emerging therapeutic opportunities. Nat Rev Drug Discov 12, 347–357, doi: 10.1038/nrd3978 (2013). 23584393

[b7] Melo S. A. . Glypican-1 identifies cancer exosomes and detects early pancreatic cancer. Nature 523, 177–182, doi: 10.1038/nature14581 (2015). 26106858 PMC4825698

[b8] Tkach M. & Théry C. Communication by Extracellular Vesicles: Where We Are and Where We Need to Go. Cell 164, 1226–1232, doi: 10.1016/j.cell.2016.01.043 (2016). 26967288

[b9] Pucci F. . SCS macrophages suppress melanoma by restricting tumor-derived vesicle-B cell interactions. Science 352, 242–246, doi: 10.1126/science.aaf1328 (2016). 26989197 PMC4960636

[b10] McKiernan J. . A Novel Urine Exosome Gene Expression Assay to Predict High-grade Prostate Cancer at Initial Biopsy. JAMA Oncol, doi: 10.1001/jamaoncol.2016.0097 (2016). 27032035

[b11] Hoshino A. . Tumour exosome integrins determine organotropic metastasis. Nature 527, 329–335, doi: 10.1038/nature15756 (2015). 26524530 PMC4788391

[b12] Montecalvo A. . Mechanism of transfer of functional microRNAs between mouse dendritic cells via exosomes. Blood 119, 756–766, doi: 10.1182/blood-2011-02-338004 (2012). 22031862 PMC3265200

[b13] Le M. T. . miR-200-containing extracellular vesicles promote breast cancer cell metastasis. J Clin Invest 124, 5109–5128, doi: 10.1172/JCI75695 (2014). 25401471 PMC4348969

[b14] Zhang L. . Microenvironment-induced PTEN loss by exosomal microRNA primes brain metastasis outgrowth. Nature 527, 100–104, doi: 10.1038/nature15376 (2015). 26479035 PMC4819404

[b15] Harazono Y. . miR-655 Is an EMT-suppressive microRNA targeting ZEB1 and TGFBR2. PLoS One 8, e62757, doi: 10.1371/journal.pone.0062757 (2013). 23690952 PMC3653886

[b16] Ling H., Fabbri M. & Calin G. A. MicroRNAs and other non-coding RNAs as targets for anticancer drug development. Nat Rev Drug Discov 12, 847–865, doi: 10.1038/nrd4140 (2013). 24172333 PMC4548803

[b17] Valadi H. . Exosome-mediated transfer of mRNAs and microRNAs is a novel mechanism of genetic exchange between cells. Nat Cell Biol 9, 654–659, doi: 10.1038/ncb1596 (2007). 17486113

[b18] Muramatsu T. . The hypusine cascade promotes cancer progression and metastasis through the regulation of RhoA in squamous cell carcinoma. Oncogene, doi: 10.1038/onc.2016.71 (2016). 27041563

[b19] Böing A. N. . Single-step isolation of extracellular vesicles by size-exclusion chromatography. J Extracell Vesicles 3, doi: 10.3402/jev.v3.23430 (2014). PMC4159761 25279113

[b20] Yuyama K., Sun H., Mitsutake S. & Igarashi Y. Sphingolipid-modulated exosome secretion promotes clearance of amyloid-β by microglia. J Biol Chem 287, 10977–10989, doi: 10.1074/jbc.M111.324616 (2012). 22303002 PMC3322859

[b21] Ono M. . Exosomes from bone marrow mesenchymal stem cells contain a microRNA that promotes dormancy in metastatic breast cancer cells. Sci Signal **7**, ra63, doi: 10.1126/scisignal.2005231 (2014). 24985346

[b22] Zhou P. . miR-17-92 plays an oncogenic role and conveys chemo-resistance to cisplatin in human prostate cancer cells. Int J Oncol 48, 1737–1748, doi: 10.3892/ijo.2016.3392 (2016). 26891588

[b23] Wang Z. . MiR-30a-5p is induced by Wnt/β-catenin pathway and promotes glioma cell invasion by repressing NCAM. Biochem Biophys Res Commun 465, 374–380, doi: 10.1016/j.bbrc.2015.08.007 (2015). 26255203

[b24] Sun X., Charbonneau C., Wei L., Chen Q. & Terek R. M. miR-181a Targets RGS16 to Promote Chondrosarcoma Growth, Angiogenesis, and Metastasis. Mol Cancer Res 13, 1347–1357, doi: 10.1158/1541-7786.MCR-14-0697 (2015). 26013170 PMC4573256

[b25] Cittelly D. M. . Downregulation of miR-342 is associated with tamoxifen resistant breast tumors. Mol Cancer 9, 317, doi: 10.1186/1476-4598-9-317 (2010). 21172025 PMC3024251

[b26] Li S. . MicroRNA expression profiling identifies activated B cell status in chronic lymphocytic leukemia cells. PLoS One 6, e16956, doi: 10.1371/journal.pone.0016956 (2011). 21408091 PMC3050979

[b27] Wang S. . MicroRNA-1246 promotes growth and metastasis of colorectal cancer cells involving CCNG2 reduction. Mol Med Rep 13, 273–280, doi: 10.3892/mmr.2015.4557 (2016). 26573378

[b28] Yuan D. . Extracellular miR-1246 promotes lung cancer cell proliferation and enhances radioresistance by directly targeting DR5. Oncotarget, doi: 10.18632/oncotarget.9017 (2016). PMC5078045 27129166

[b29] Kojima T. . Decreased expression of CXXC4 promotes a malignant phenotype in renal cell carcinoma by activating Wnt signaling. Oncogene 28, 297–305, doi: 10.1038/onc.2008.391 (2009). 18931698

[b30] Liu W. M. . A microarray study of altered gene expression in colorectal cancer cells after treatment with immunomodulatory drugs: differences in action *in vivo* and *in vitro*. Mol Biol Rep 37, 1801–1814, doi: 10.1007/s11033-009-9614-3 (2010). 19597962

[b31] Ling B. . Suppression of non-small cell lung cancer proliferation and tumorigenicity by DENND2D. Lung Cancer 79, 104–110, doi: 10.1016/j.lungcan.2012.10.012 (2013). 23182661

[b32] Baranyai T. . Isolation of Exosomes from Blood Plasma: Qualitative and Quantitative Comparison of Ultracentrifugation and Size Exclusion Chromatography Methods. PLoS One 10, e0145686, doi: 10.1371/journal.pone.0145686 (2015). 26690353 PMC4686892

[b33] Hong C. S., Funk S., Muller L., Boyiadzis M. & Whiteside T. L. Isolation of biologically active and morphologically intact exosomes from plasma of patients with cancer. J Extracell Vesicles 5, 29289 (2016). 27018366 10.3402/jev.v5.29289 PMC4808740

[b34] Peinado H. . Melanoma exosomes educate bone marrow progenitor cells toward a pro-metastatic phenotype through MET. Nat Med 18, 883–891, doi: 10.1038/nm.2753 (2012). 22635005 PMC3645291

[b35] Tominaga N. . Brain metastatic cancer cells release microRNA-181c-containing extracellular vesicles capable of destructing blood-brain barrier. Nat Commun 6, 6716, doi: 10.1038/ncomms7716 (2015). 25828099 PMC4396394

[b36] Zekri A. R. . Circulating Serum miRNAs as Diagnostic Markers for Colorectal Cancer. PLoS One 11, e0154130, doi: 10.1371/journal.pone.0154130 (2016). 27135244 PMC4852935

[b37] Braicu C. . Exosomes as divine messengers: are they the Hermes of modern molecular oncology? Cell Death Differ 22, 34–45, doi: 10.1038/cdd.2014.130 (2015). 25236394 PMC4262777

[b38] Zhao L. & Zhang Y. miR-342-3p affects hepatocellular carcinoma cell proliferation via regulating NF-κB pathway. Biochem Biophys Res Commun 457, 370–377, doi: 10.1016/j.bbrc.2014.12.119 (2015). 25580008

[b39] Hibino S. . Reduced expression of DENND2D through promoter hypermethylation is an adverse prognostic factor in squamous cell carcinoma of the esophagus. Oncol Rep 31, 693–700, doi: 10.3892/or.2013.2901 (2014). 24317529

[b40] Zhang T. . Downregulation of miR-522 suppresses proliferation and metastasis of non-small cell lung cancer cells by directly targeting DENN/MADD domain containing 2D. Sci Rep 6, 19346, doi: 10.1038/srep19346 (2016). 26783084 PMC4726064

[b41] Muramatsu T. . YAP is a candidate oncogene for esophageal squamous cell carcinoma. Carcinogenesis 32, 389–398, doi: 10.1093/carcin/bgq254 (2011). 21112960

[b42] Fujiwara N. . miR-634 Activates the Mitochondrial Apoptosis Pathway and Enhances Chemotherapy-Induced Cytotoxicity. Cancer Res 75, 3890–3901, doi: 10.1158/0008-5472.CAN-15-0257 (2015). 26216549

