# Supplementary material for: Exosomal microRNA miR-1246 induces cell motility and invasion through the regulation of DENND2D in oral squamous cell carcinoma
Source: Sci Rep. 2016 Dec 8;6:38750. doi: 10.1038/srep38750 (PMC5144099; doi:10.1038/srep38750)

Submission to *Scientific Reports*

Exosomal microRNA, *miR-1246*, induces cell motility and invasion through the regulation of *DENND2D* in oral squamous cell carcinoma

Sujata Sakha D.D.S.[1], Tomoki Muramatsu, Ph.D.[1], Koji Ueda, Ph.D.[3], and Johji Inazawa, M.D., Ph.D.[1,2]

[1]Department of Molecular Cytogenetics, Medical Research Institute, Tokyo Medical and Dental University, Tokyo, Japan. [2]Bioresource Research Center, Tokyo Medical and Dental University, Tokyo, Japan. [3]Project for Personalized Cancer Medicine, Genome Center, Japanese Foundation for Cancer Research, Tokyo, Japan

Supplementary Information

Supplementary Figure S1. Analysis of RNA from cells and exosomes isolated by the size-exclusion chromatography method using an Agilent Bioanalyzer.

Supplementary Figure S2. LM-exosomes do not affect the cell growth, migration and invasion abilities of HOC313-LM cells.

Supplementary Figure S3. Extraction of candidate miRNAs as oncomiRs.

Supplementary Figure S4. *MiR-1246* promotes cell migration and invasion through the regulation of DENND2D in TSU cells.

Supplementary Figure S5. *MiR-1246* promotes cell migration and invasion through the regulation of DENND2D in HeLa cells

Supplementary Figure S6. Analysis of RNA from LM-exosomes isolated by ultracentrifugation and by an exosome isolation kit using an Agilent Bioanalyzer.

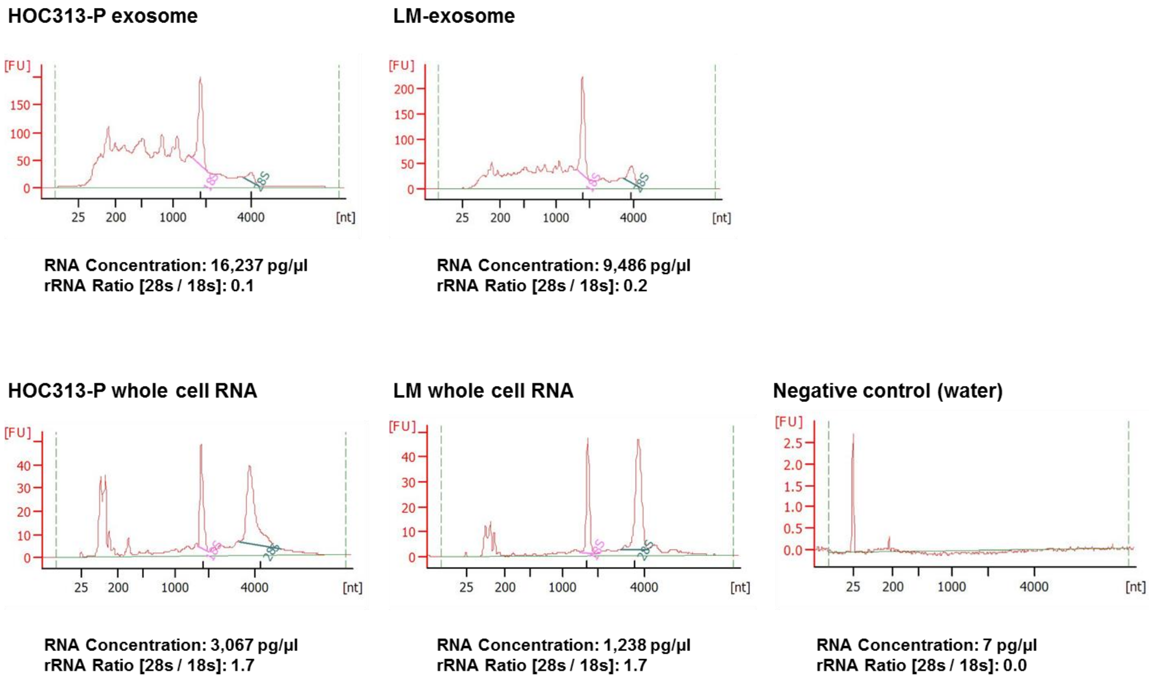

Supplementary Figure S1. **Analysis of RNA from cells and exosomes isolated by the size-exclusion chromatography method using an Agilent Bioanalyzer.**

Total RNA was isolated from whole cells and exosomes in the both of HOC313-P and -LM cells and RNA quality was analyzed by Agilent 2100 Bioanalyzer. The 18S and 28S rRNA were dominant peaks in RNA from whole cells. nt: nucleotide length. FU: fluorescent units.

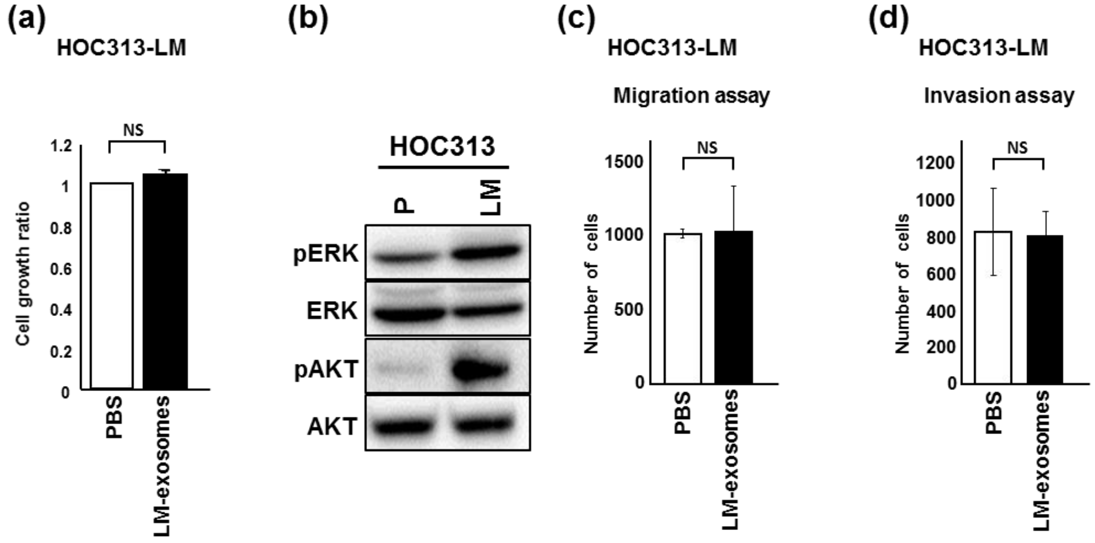

Supplementary Figure S2. LM-exosomes do not affect the cell growth, migration and invasion abilities of HOC313-LM cells.

(a) The effect of LM-exosomes on cell proliferation of HOC313-LM was determined by WST-8 assay after incubation with PBS or LM-exosomes for 5 days. The growth was not affected. (b) Western blotting analysis of ERK and AKT and their phosphorylation status were shown in HOC313-P and -LM cells. (c, d) Cell motility was assessed by transwell migration assay (c) and cell invasion by transwell invasion assay (d) after treatment of PBS or LM-exosomes in HOC313-LM cells. Experiments were repeated for three times. (Bars, SD). The student t-test was used for statistical analysis. NS: no significance.

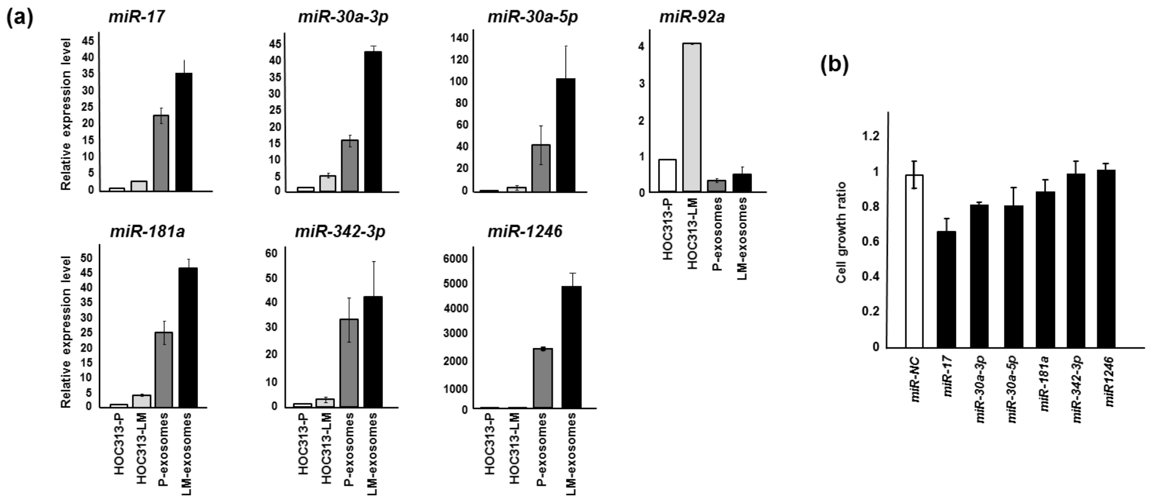

Supplementary Figure S3. Extraction of candidate miRNAs as oncomiRs.

(a) Validation of candidate miRNA expression in HOC313-P and -LM cell as well as their respective exosomes was measured by qRT-PCR. Their expressions were normalized by *RNU6B* expression. The expression of *miR-92a* was not validated by qRT-PCR. (b) The cell growth after five days of treatment with these miRNAs or negative control in HOC313-P cells was assessed by WST-8 assay. Each data point represents the mean of three experiments. (bars, SD).

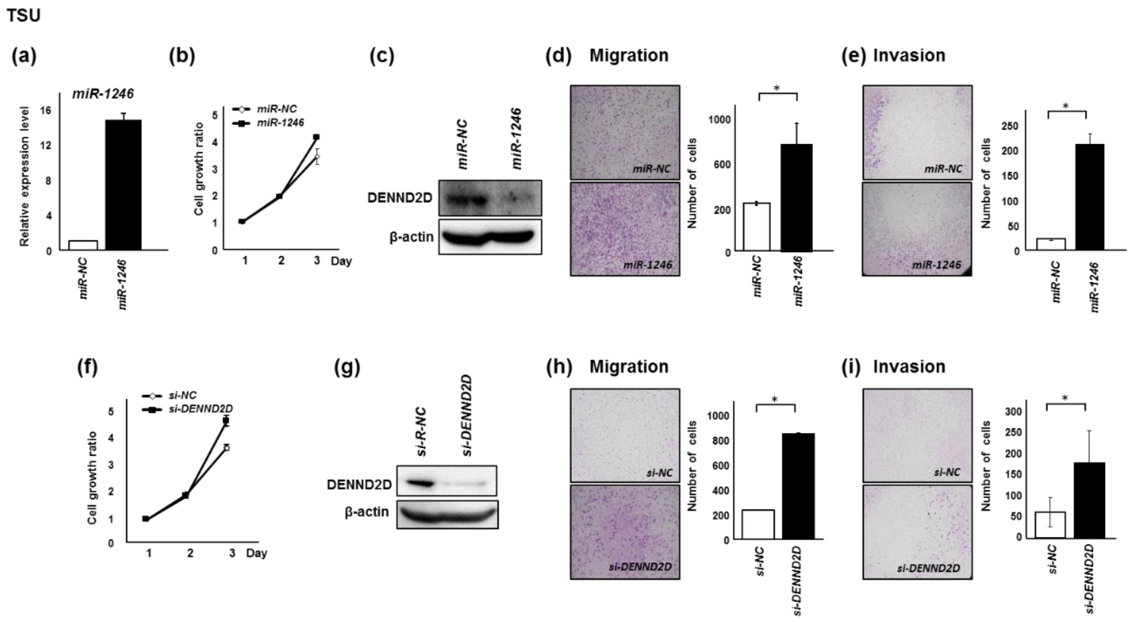

Supplementary Fig. S4. ***MiR-1246*** **promotes cell migration and invasion through the regulation of** ***DENND2D*** **in TSU cells.**

(a) Validation of *miR-1246* expression after miRNA transfection in TSU cells was measured by qRT-PCR. The expression of *miR-1246* was normalized by RNU6B expression. (b, f) The effect of *miR-1246* (b) or DENND2D-specific siRNA (f) on cell proliferation of TSU cells was evaluated by WST-8 assay. (c, g) Downregulation of DENND2D at the protein level was confirmed by western blot analysis after transfection of miR-1246 (c) or DENND2D-specific siRNA (g). (d-e, h-i) Cell migration was assessed by transwell migration assay (d, h) and cell invasion by transwell invasion assay (e, i) with *miR-1246* (d-e) or DENND2D-specific siRNA (h-i) transfection in TSU cells.

Experiments were performed in triplicate. (Bars, SD). The student *t*-test was used for statistical analysis; asterisks represent $P<0.05$ versus each control transfectant.

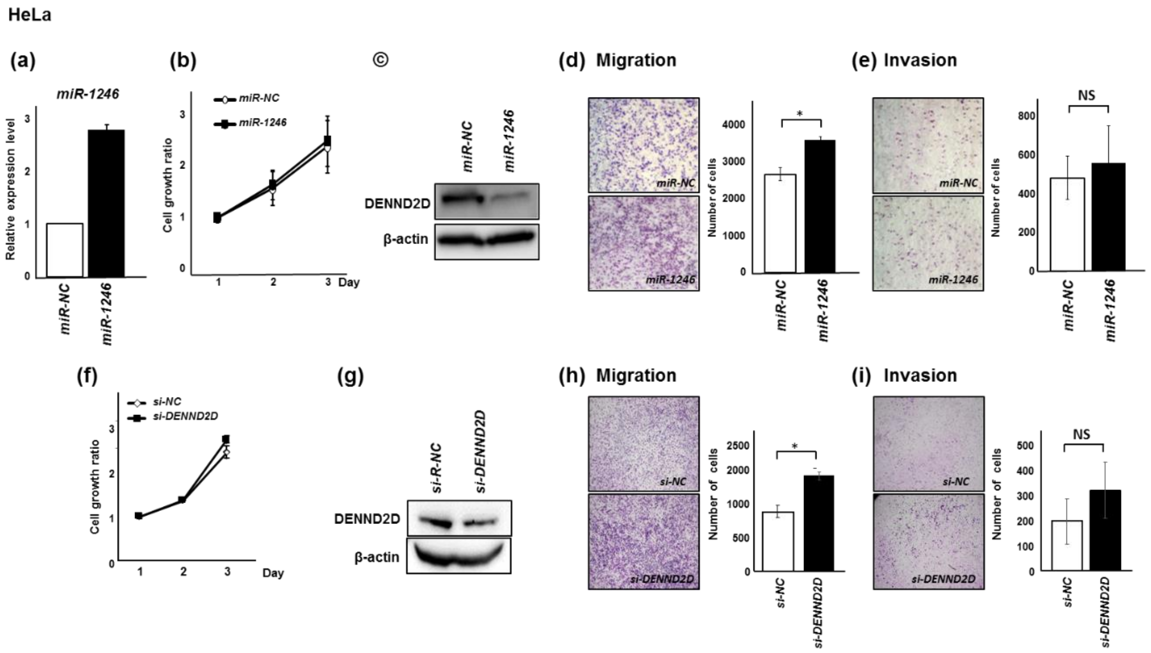

Supplementary Fig. S5. *MiR-1246* promotes cell migration and invasion through the regulation of *DENND2D* in HeLa cells.

(a) Validation of *miR-1246* expression after miRNA transfection in HeLa cells was measured by qRT-PCR. The expression of *miR-1246* was normalized by RNU6B expression. (b, f) The effect of *miR-1246* (b) or DENND2D-specific siRNA (f) on cell proliferation of HeLa cells was evaluated by WST-8 assay. (c, g) The expression of DENND2D was analyzed by western blotting analysis in HeLa cells after transfection with miR-1246 or DENND2D-specific siRNA for 72 hours. (d-e, h-i) Cell migration was assessed by transwell migration assay (d, h) and cell invasion by transwell invasion assay (e, i) with *miR-1246 (d-e)* or DENND2D-specific siRNA (h-i) transfection in HeLa

cells. Experiments were performed in triplicate. (Bars, SD). The student *t*-test was used for statistical analysis; asterisks represent $P<0.05$ versus each control transfectant.

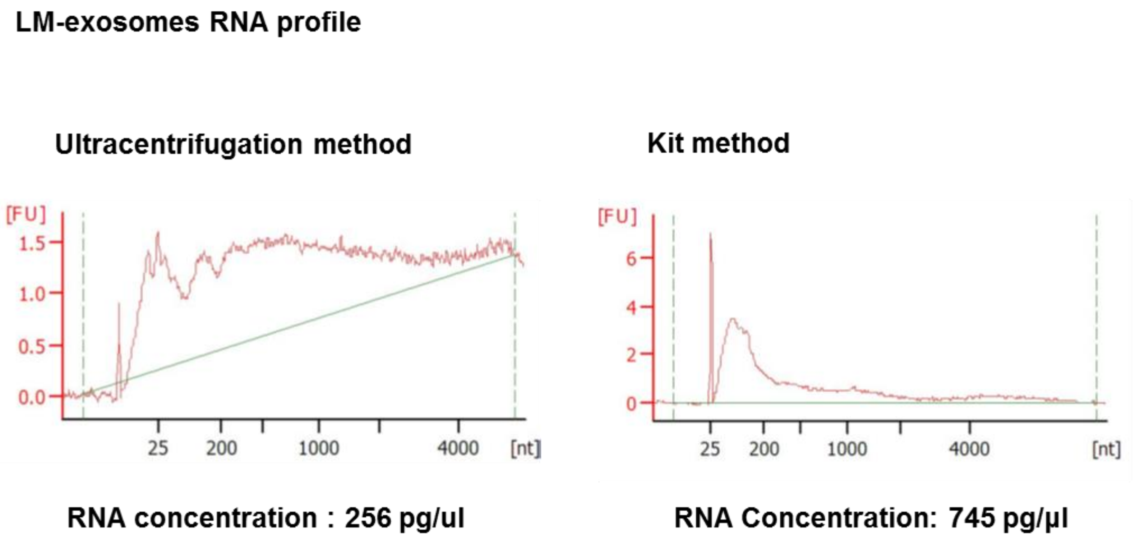

Supplementary Figure S6. Analysis of RNA from LM-exosomes isolated by ultracentrifugation and by an exosome isolation kit using an Agilent Bioanalyzer.

Total RNA was isolated from exosomes of HOC313-LM cells in ultracentrifugation and kit methods and RNA quality was analyzed by Agilent 2100 Bioanalyzer. nt: nucleotide length. FU: fluorescent units.

Figure 1b

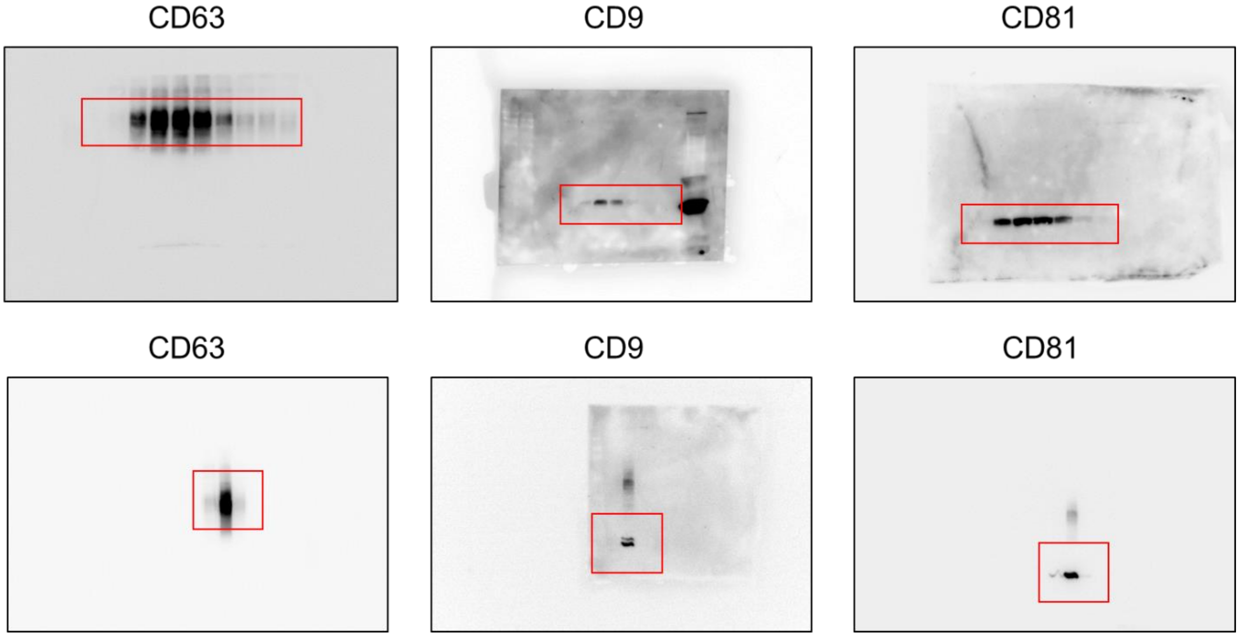

Figure 2b

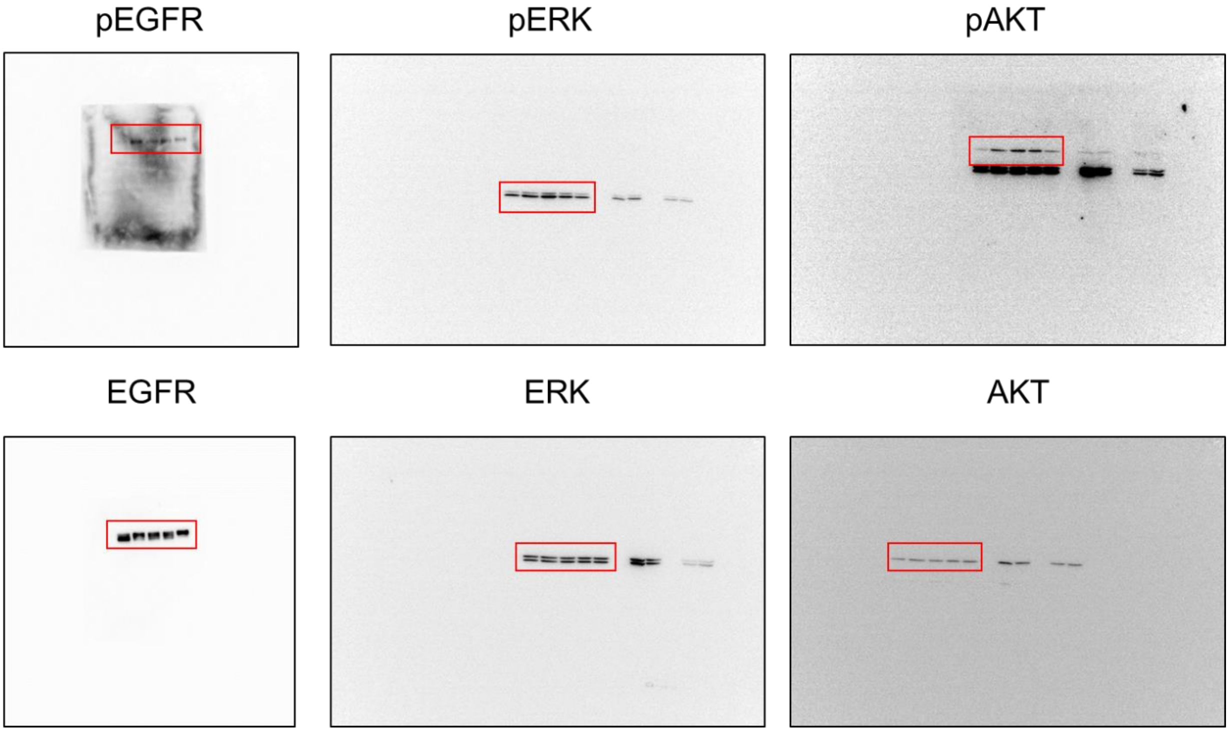

Figure 4b

DENND2D

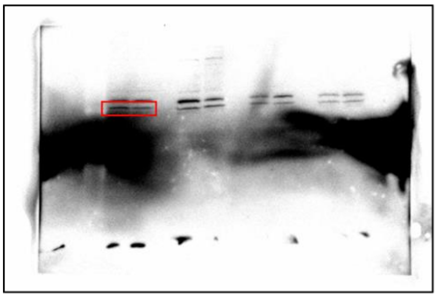

DENND2D

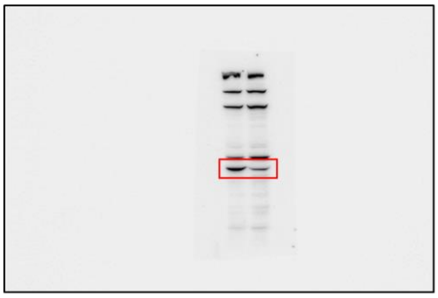

Figure 4c

DENND2D

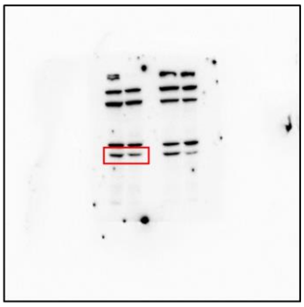

β-actin

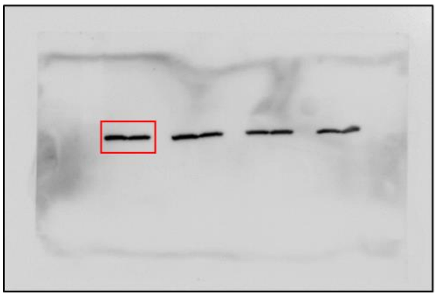

β-actin

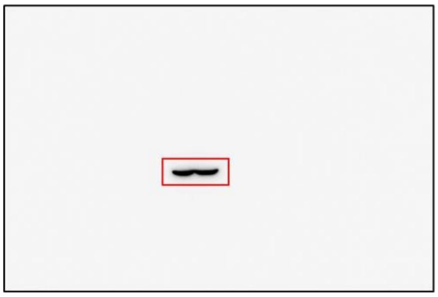

β-actin

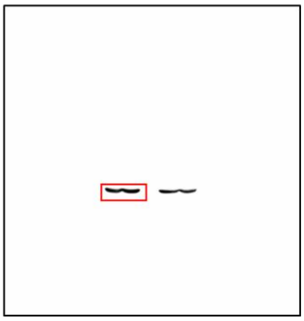

Supplementary Figure 2Sb

pERK

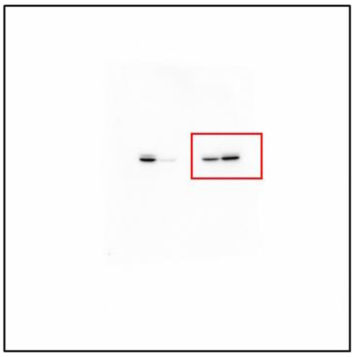

pAKT

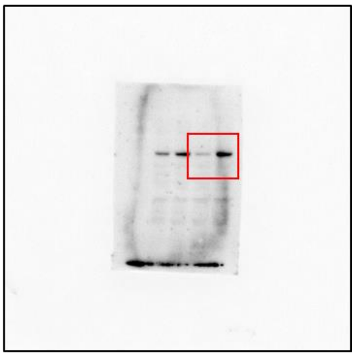

ERK

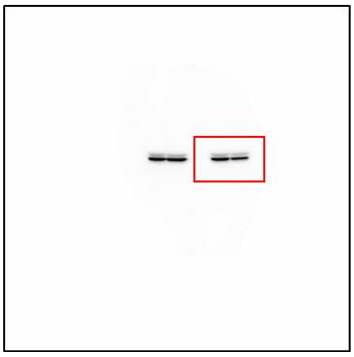

AKT

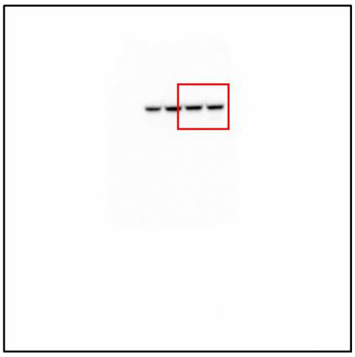

Supplementary Figure 4Se

DENND2D

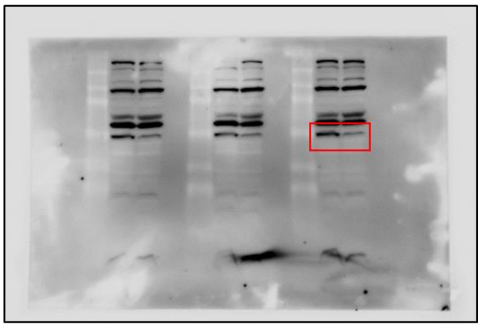

β-actin

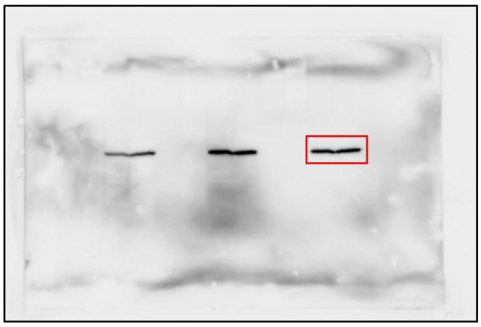

Supplement: Supplementary Information [file srep38750-s1.pdf]
